# Supplementary material for: Cognitive load as a mediator in self-efficacy and English learning motivation among vocational college students
Source: PLoS One. 2024 Nov 21;19(11):e0314088. doi: 10.1371/journal.pone.0314088 (PMC11581323; doi:10.1371/journal.pone.0314088)
Supplement: S1 File — (DOCX) [file pone.0314088.s001.docx]

**Title**

Cognitive Load as a Mediator in Self-Efficacy and English Learning Motivation among Vocational College Students

**Statements and Declarations**

**A Data Availability Statement**

To ensure the validity of the responses, some questions were specially designed to determine whether the participant was answering carefully or not. For example, two similar questions would appear in the questionnaire but required an opposite answer. If the participant was not paying careful attention and answering intently, they would provide the same answer for each question, and this would result in their data being excluded from the final collection. Data collection lasted for one week during June 21-27, 2023. Exclusion criteria regarding the responses included the following: (1) if the answers to multiple questions were the same or close; (2) if a participant failed the polygraph test (inconsistencies or answers that did not make sense).

The study randomly selected a total of 200 college students from XX University and XX Vocational College, with 100 from each school. Among them, 75 were boys and 125 were girls. A questionnaire survey was conducted using the questionnaire platform. A total of 200 questionnaires were distributed, and 168 valid questionnaires were collected with an effective recovery rate of 84%.

We confirm that the experimental protocol was approved by academic committee of the school.

The datasets analyzed during the current study are available from the corresponding author on reasonable request. All data analyzed during this study are included in this published article. No conflict of interest

**Consent to Participate Declaration**

I voluntarily participate in the research *Cognitive Load as a Mediator in Self-Efficacy and English Learning Motivation among Vocational College Students*. I understand the purpose, methods, potential risks, and benefits of this research. Prior to participating in the research, I have received sufficient information and had the opportunity to ask questions and receive satisfactory answers from the research team.

I agree to participate in this research based on my own free choice. I understand that during the research, I will comply with all research protocols and instructions provided by the researchers. I understand and agree that after the research is completed, the research team may share my data and results, without disclosing my personal information.

By signing this declaration, I indicate that I agree to the above terms and that I am willing to participate in this research study.

All the participants confirm the above consent before doing the questionnaire.

**Statement of Compliance with Ethical Principles**

We state that our research complies with ethical principles, including respect for personal dignity and privacy, ensuring informed consent, fairness and equity, adherence to academic norms, and integrity.

We state that we comply with relevant laws, regulations, and ethical norms, such as the Declaration of Helsinki, to ensure the legality and ethics of the research.

We state that we will accept ethical review and supervision to ensure the ethical and scientific nature of the research.

Our research strictly adheres to the regulations set forth by the academic research institution of our school and is fully subject to its supervision and guidance.
