# Supplementary material for: Cognitive load as a mediator in self-efficacy and English learning motivation among vocational college students
Source: PLoS One. 2024 Nov 21;19(11):e0314088. doi: 10.1371/journal.pone.0314088 (PMC11581323; doi:10.1371/journal.pone.0314088)
Supplement: Attachment — Submitted filename: renamed_a74de.docx [file pone.0314088.s002.docx]

Dear editor,

I would like to express my sincere gratitude for the opportunity to revise my manuscript and for the constructive feedback you have provided. Below, please find my responses to the reviewer. I hope that these revisions meet the expectations of the journal and its readership.

1. Please ensure that your manuscript meets PLOS ONE's style requirements, including those for file naming. The PLOS ONE style templates can be found at

https://journals.plos.org/plosone/s/file?id=wjVg/PLOSOne_formatting_sample_main_body.pdf and

https://journals.plos.org/plosone/s/file?id=ba62/PLOSOne_formatting_sample_title_authors_affiliations.pdf

**Answer:**

I have carefully reviewed and updated the manuscript to ensure it fully complies with PLOS ONE's style requirements, including adhering to the guidelines for file naming conventions

2. You indicated that ethical approval was not necessary for your study. We understand that the framework for ethical oversight requirements for studies of this type may differ depending on the setting and we would appreciate some further clarification regarding your research. Could you please provide further details on why your study is exempt from the need for approval and confirmation from your institutional review board or research ethics committee (e.g., in the form of a letter or email correspondence) that ethics review was not necessary for this study? Please include a copy of the correspondence as an ""Other"" file.

**Answer:**

The ethical statement has already been provided in the document titled "Supporting Information."

3. We note that you have indicated that there are restrictions to data sharing for this study. PLOS only allows data to be available upon request if there are legal or ethical restrictions on sharing data publicly. For more information on unacceptable data access restrictions, please see http://journals.plos.org/plosone/s/data-availability#loc-unacceptable-data-access-restrictions.

Before we proceed with your manuscript, please address the following prompts:

a) If there are ethical or legal restrictions on sharing a de-identified data set, please explain them in detail (e.g., data contain potentially identifying or sensitive patient information, data are owned by a third-party organization, etc.) and who has imposed them (e.g., a Research Ethics Committee or Institutional Review Board, etc.). Please also provide contact information for a data access committee, ethics committee, or other institutional body to which data requests may be sent.

b) If there are no restrictions, please upload the minimal anonymized data set necessary to replicate your study findings to a stable, public repository and provide us with the relevant URLs, DOIs, or accession numbers. For a list of recommended repositories, please see

https://journals.plos.org/plosone/s/recommended-repositories. You also have the option of uploading the data as Supporting Information files, but we would recommend depositing data directly to a data repository if possible.

We will update your Data Availability statement on your behalf to reflect the information you provide.

**Answer:**

In compliance with PLOS ONE's guidelines, the minimal anonymized dataset necessary to replicate the study findings has been successfully uploaded to a stable, public repository. Additionally, the Mplus code used in our analysis has also been deposited. The relevant URLs and accession numbers for the datasets and code are provided below

https://www.openicpsr.org/openicpsr/project/204782/version/V1/view

4. Please include your full ethics statement in the ‘Methods’ section of your manuscript file. In your statement, please include the full name of the IRB or ethics committee who approved or waived your study, as well as whether or not you obtained informed written or verbal consent. If consent was waived for your study, please include this information in your statement as well.

**Answer:**

A comprehensive ethics statement has been incorporated into the 'Methods' section of the manuscript, aligning with the journal's guidelines. The updated section now details the following:

The study was conducted in strict adherence to the ethical guidelines and standards for research involving human subjects, as stipulated by the laws and regulations in China. The study design underwent a thorough review and received approval from the relevant ethics review board., ensuring that the research was conducted with the utmost respect for the rights, safety, and well-being of all participants. Informed consent was obtained from all participants after providing them with a thorough explanation of the study's objectives, procedures, potential risks, benefits, and their right to withdraw at any time without any adverse consequences. Documentation of consent was secured, ensuring compliance with the principles of voluntary participation and confidentiality.

5. Please include a separate caption for each figure in your manuscript.

**Answer:**

Captions for all figures have been incorporated into the manuscript as per the journal's guidelines

6. Please review your reference list to ensure that it is complete and correct. If you have cited papers that have been retracted, please include the rationale for doing so in the manuscript text, or remove these references and replace them with relevant current references. Any changes to the reference list should be mentioned in the rebuttal letter that accompanies your revised manuscript. If you need to cite a retracted article, indicate the article’s retracted status in the References list and also include a citation and full reference for the retraction notice.

**Answer:**

The reference list has been reviewed and updated.
