# Supplementary material for: Cognitive load as a mediator in self-efficacy and English learning motivation among vocational college students
Source: PLoS One. 2024 Nov 21;19(11):e0314088. doi: 10.1371/journal.pone.0314088 (PMC11581323; doi:10.1371/journal.pone.0314088)
Supplement: Attachment — Submitted filename: Cognitive Load as a Mediator in Self.docx [file pone.0314088.s003.docx]

**Cognitive Load as a Mediator in Self-Efficacy and English Learning Motivation among Vocational**

**College Students
Reviewer Comments:**

1. The introduction fails to comprehensively attend to and expand the research concerns.

2. There exist limited references despite the vast array of contemporary studies on self-efficacy. Hence, further probing into the literature is required.

3. Font concordance throughout the text is missing.

4. A few grammatical issues exist.

5. In section one of the literature review (hypothesis 1), studies on self-efficacy and its principal value in the educational context are not addressed. Most studies have self-efficacy as a probable byproduct of a method or teaching approach; nevertheless, the concept of self-efficacy as the core content of research is well-established yet not addressed by the current author/researcher.

6. The literature review is fragmented, with sectionalized hypotheses. Additionally, this section needs augmentation.

7. An excessive number of hypotheses are raised for a single article. Consequently, the focused scope and undivided attention to the research aims are disturbed. Moreover, the approach taken toward the concept proposed is not in congruence with the hypotheses.

8. Hypotheses for section 1 are directional; however, some studies do not support self-efficacy as the predictive factor of motivation. Moreover, the literature review is too confined to yield four hypotheses. The author has taken many facts for granted, evidenced by the number of hypotheses (13 in total, namely four major and nine minor) not leveled with the number of references and studies recapitulated in terms of quality and quantity.

9. According to the literature review (section 2; hypothesis 2), self-efficacy predicts cognitive load; how is the “cognitive load as a mediator in self-efficacy” justified?

10. The literature review needs an in-depth analysis of studies associated with the current research scope.

11. The methodology must be delineated and clarified with more detail and descriptions.

12. The proposed title is not in concert with the research as the synergy of self-efficacy, motivation, and cognitive load are investigated rather than the absolute mediating effect of cognitive load on the dependent and independent variables.

13. Theoretical implications: how are your research findings and claims in concert with Shao and Kang’s “parent-child relationship” concerns?

14. Findings need to be further discussed and compared with similar research findings.

15. Practical implications: AI’s incorporation is not well-chained to the findings and implications of the current study.
