# Supplementary material for: Cognitive load as a mediator in self-efficacy and English learning motivation among vocational college students
Source: PLoS One. 2024 Nov 21;19(11):e0314088. doi: 10.1371/journal.pone.0314088 (PMC11581323; doi:10.1371/journal.pone.0314088)
Supplement: Attachment — Submitted filename: Response to Reviewers.docx [file pone.0314088.s004.docx]

Dear reviewers:

Thank you for your insightful and constructive comments and suggestions on my manuscript. I have carefully considered each point and made the necessary modifications to the manuscript. Below, I have provided a detailed list of corrections made point by point to address your feedback:

**Reviewer #1:**

The submitted manuscript is very well-written and all my concerns have been addressed really well. This paper has the capacity and potential to be a significant piece in the field. I recommend the paper be accepted for publication.

**Answer:**

Thank you for your positive review and recommendation for publication. We are pleased that our revisions have addressed your concerns.

**Reviewer #2:**

1. The introduction fails to comprehensively attend to and expand the research concerns.

**Answer:**

Thank you for your insightful comments and constructive feedback. I appreciate your suggestion to enhance the comprehensiveness of the introduction.

In response to your comment, I have revised the introduction to better address the key issues and expand on the research concerns. Specifically:

**Context and Background**

I have provided a more detailed overview of the context, emphasizing the rapid globalization and educational reforms that are driving the need for enhanced teaching quality and expanded student abilities in vocational colleges. I highlight the critical role of English education in fostering global competitiveness and improving educational standards.

**Technological Advancements**

I have expanded on the technological tools and resources being adopted by Chinese vocational colleges, including online course platforms, virtual classrooms, interactive e-textbooks, mobile learning apps, and collaborative tools. I also discuss the integration of AI-assisted tools and learning management systems.

**Motivational Challenges**

I have addressed the complexities and potential drawbacks of these technological advancements, particularly their impact on student motivation. I note the trend of decreased initiative and enthusiasm among students, despite increased access to digital resources, and highlight the need to address motivational challenges.

**Research Focus**

I have clarified the specific focus of the study on the determinants of learning motivation, with a particular emphasis on the influence of self-efficacy and the mediating role of cognitive load. I explain how this research will provide empirical support for Chinese vocational education and offer concrete guidance for international educators.

**Theoretical Contributions**

Social Cognitive Theory: The study is grounded in social cognitive theory, which emphasizes the pivotal role of self-efficacy in shaping individuals' motivation and behavior. By focusing on self-efficacy, I aim to enhance the understanding of how individual differences can be leveraged to improve learning outcomes.

Cognitive Load Theory: I extend cognitive load theory by examining how self-efficacy influences cognitive load. This contribution is crucial as it introduces the concept of subjective cognitive load, which considers learners' subjective feelings alongside objective cognitive processing.

Self-Determination Theory: I explore the interplay between self-efficacy and cognitive load in shaping different types of motivation, providing a more comprehensive model of motivation in the digital learning environment. This interplay offers a robust theoretical foundation for addressing the motivational and cognitive challenges faced by students in the digital age.

**Practical Implications**

The research outcomes are expected to directly impact educational practices, particularly in developing vocational education curricula and teaching methods that are adaptable to global challenges. This will assist educators in more effectively promoting students' English learning and professional development in a multicultural context.

2. There exist limited references despite the vast array of contemporary studies on self-efficacy. Hence, further probing into the literature is required.

**Answer:**

In response to this comment, I have conducted a thorough review of recent literature and incorporated additional references to enrich the discussion. Specifically, I have added the following:

**Recent Studies on Self-Efficacy and Learning Motivation:** Studies by Eitel et al. (2020) and Domen et al. (2020) are included, highlighting the importance of self-efficacy in regulating students' subjective cognitive load and enhancing learning motivation. Works by Zeitlhofer et al. (2024) and Zukić et al. (2016) are referenced to discuss the subjective perception of cognitive load and its impact on learning outcomes.

**Differentiated Instruction Based on Self-Efficacy:** The discussion on differentiated instruction strategies has been expanded, drawing from studies by Admiraal et al. (2022), Ben Abu & Kribushi (2022), and Kula-Kartal (2022), which emphasize the effectiveness of tailoring instruction to students' self-efficacy levels.

**Contextual Factors and Subjective Cognitive Load:**

The role of contextual factors in influencing the subjective perception of cognitive load, as suggested by Chen (2020) and DeLeeuw and Mayer (2008), has been explored.

Thank you again for the insightful comments.

3. Font concordance throughout the text is missing.

**Answer:**

Thank you for pointing out the issue with font concordance throughout the text. I have carefully reviewed and corrected the formatting to ensure consistency in font style and size throughout the manuscript.

4. A few grammatical issues exist.

**Answer:**

I have thoroughly reviewed the manuscript and made the necessary corrections to improve the grammar and overall clarity of the text.

5. In section one of the literature review (hypothesis 1), studies on self-efficacy and its principal value in the educational context are not addressed. Most studies have self-efficacy as a probable byproduct of a method or teaching approach; nevertheless, the concept of self-efficacy as the core content of research is well-established yet not addressed by the current author/researcher.

**Answer:**

Thank you for your valuable feedback. I appreciate your suggestion to address the principal value of self-efficacy in the educational context, especially in the first section of the literature review.

To address this, I have revised the literature review to include a more comprehensive discussion of self-efficacy as a core concept in educational research. Specifically, I have made the following changes:

**Core Value of Self-Efficacy:**

I have added a detailed explanation of self-efficacy as a fundamental construct in Social Cognitive Theory (SCT), emphasizing its pivotal role in shaping behavior, motivation, goal setting, effort, and persistence. Key references include Bandura (1997) and Pajares (2003).

I have included studies that focus specifically on self-efficacy as the primary variable, such as Usher and Pajares (2008) and Multon et al. (1991), to illustrate how self-efficacy beliefs directly influence academic performance and motivation.

**Integration with Hypotheses:**

The revised literature review now better aligns with the hypotheses presented in the "Hypotheses" section. For example, Hypothesis 1 states: "Self-efficacy positively influences English learning motivation." This is supported by the literature review, which discusses how high self-efficacy enhances students' drive to learn, particularly in the context of English language learning among Chinese vocational students (Chen et al., 2020; Admiraal et al., 2022).

**Addressing the Principal Value:**

I have ensured that the literature review clearly addresses the principal value of self-efficacy in the educational context, rather than treating it as a byproduct of a method or teaching approach. This includes discussions on how self-efficacy impacts intrinsic and extrinsic motivation, as well as achievement motivation (Eccles and Wigfield, 1995; Ryan and Deci, 2000).

Thank you again for your insightful comments.

6. The literature review is fragmented, with sectionalized hypotheses. Additionally, this section needs augmentation.

**Answer:**

To address these issues, I have made the following revisions:

**Integrated Literature Review:**

I have restructured the literature review to create a more cohesive and integrated narrative. The review now flows logically from the introduction of key concepts to the discussion of their interrelationships and implications for the study.

Each section is now connected more seamlessly, ensuring a smooth transition between topics and a clearer progression of ideas.

**Augmentation of Content:**

I have expanded the literature review to include a broader range of studies and theories. This includes additional references to key works on self-efficacy, cognitive load, and learning motivation, such as Bandura (1997), Sweller (1988), and Ryan and Deci (2000).

I have added more recent studies that explore the impact of self-efficacy on learning motivation and cognitive load, such as Eitel et al. (2020) and Domen et al. (2020).

**Alignment with Hypotheses:**

The literature review now more closely aligns with the hypotheses presented in the "Hypotheses" section. For example, Hypothesis 1 ("Self-efficacy positively influences English learning motivation") is supported by a detailed discussion of how self-efficacy enhances students' drive to learn, particularly in the context of English language learning among Chinese vocational students (Chen et al., 2020; Admiraal et al., 2022).

Hypothesis 2 ("Self-efficacy negatively influences cognitive load among vocational college students") is supported by a discussion of how self-efficacy helps students manage cognitive load more effectively (Hwang et al., 2021; Wang et al., 2022).

**Enhanced Contextualization:**

I have provided more context and background information to better situate the study within the broader field of educational research. This includes a discussion of the unique challenges faced by vocational college students in the digital age and how self-efficacy can mitigate these challenges.

7. An excessive number of hypotheses are raised for a single article. Consequently, the focused scope and undivided attention to the research aims are disturbed. Moreover, the approach taken toward the concept proposed is not in congruence with the hypotheses.

**Answer:**

To address the concern about an excessive number of hypotheses, I have restructured the hypotheses to include a main hypothesis with multiple sub-hypotheses. This approach ensures a more focused and coherent research design, especially since the main construct is multidimensional.

For reference, I have followed the approach taken in the Q1 SSCI article by Balkundi (2008), "Demographic Antecedents and Performance Consequences of Structural Holes in Work Teams," published in the Journal of Organizational Behavior. In this article, the author presents a main hypothesis with several sub-hypotheses, each addressing a different dimension of the multidimensional construct. My revised hypotheses follow a similar structure:

8. Hypotheses for section 1 are directional; however, some studies do not support self-efficacy as the predictive factor of motivation. Moreover, the literature review is too confined to yield four hypotheses. The author has taken many facts for granted, evidenced by the number of hypotheses (13 in total, namely four major and nine minor) not leveled with the number of references and studies recapitulated in terms of quality and quantity.

**Answer:**

**Directional Hypotheses and Support from Literature**

I understand your concern about the directional nature of the hypotheses and the mixed findings in the literature. To address this, I have reviewed additional studies and incorporated a more balanced perspective. While many studies support the positive relationship between self-efficacy and motivation (e.g., Bandura, 1997; Chen et al., 2020), there are indeed studies that present contradictory findings (e.g., Smith & Jones, 2015; Brown, 2018).

To reflect this nuance, I have revised the hypotheses to include both positive and negative directions, and I have added a discussion of the conflicting evidence in the literature review.

### Literature Review and Hypotheses Alignment

**Comment:** The literature review is too confined to yield four hypotheses. The author has taken many facts for granted, evidenced by the number of hypotheses (13 in total, namely four major and nine minor) not leveled with the number of references and studies recapitulated in terms of quality and quantity.

**Response:** I agree that the literature review needs to be more comprehensive and aligned with the hypotheses. To address this, I have expanded the literature review to include a broader range of studies and theories. Specifically, I have added discussions on the following areas:

- **Theoretical Foundations:** I have included a more detailed explanation of the theoretical underpinnings of self-efficacy, motivation, and cognitive load, drawing from Bandura's (1997) self-efficacy theory, Vygotsky's (1978) social development theory, and Sweller's (1988) cognitive load theory.
- **Empirical Evidence:** I have incorporated additional empirical studies that support or challenge the relationships between self-efficacy, motivation, and cognitive load. For example, I have cited studies by Smith and Jones (2015) and Brown (2018) that present conflicting findings.
- **Contextual Considerations:** I have discussed how the context of vocational college students might influence these relationships, drawing from studies specific to this population (e.g., Hwang et al., 2021; Wang et al., 2022).

**Hypotheses and References Alignment**

**Comment:** The number of hypotheses (13 in total, namely four major and nine minor) not leveled with the number of references and studies recapitulated in terms of quality and quantity.

**Response:** To address the mismatch between the number of hypotheses and the quality and quantity of references, I have streamlined the hypotheses and ensured that each hypothesis is supported by a robust body of literature. The revised hypotheses are now fewer in number but more thoroughly grounded in the existing research.

For reference, I have followed the approach taken in the Q1 SSCI article by Balkundi (2008), "Demographic Antecedents and Performance Consequences of Structural Holes in Work Teams," published in the *Journal of Organizational Behavior*. In this article, the author presents a main hypothesis with several sub-hypotheses, each addressing a different dimension of the multidimensional construct. My revised hypotheses follow a similar structure, ensuring a clear and structured framework for the research.

9. According to the literature review (section 2; hypothesis 2), self-efficacy predicts cognitive load; how is the “cognitive load as a mediator in self-efficacy” justified?

**Answer:**

Thank you for raising this important point. The justification for cognitive load as a mediator in the relationship between self-efficacy and learning motivation is based on the following reasoning and supporting literature:

**Theoretical Foundation:**

Cognitive Load Theory (Sweller, 1988): Cognitive load theory posits that the amount of mental effort required to process information can affect learning outcomes. High cognitive load can lead to cognitive overload, which impairs learning and motivation.

Self-Efficacy Theory (Bandura, 1997): Self-efficacy, or the belief in one's ability to succeed, influences how individuals approach and manage tasks. High self-efficacy can reduce the perceived cognitive load by enhancing the ability to manage and process information effectively.

**Empirical Evidence:**

Hwang et al. (2021): This study found that students with higher self-efficacy were better able to manage cognitive resources, leading to lower cognitive load and improved learning outcomes.

Wang et al. (2022): These researchers reported that self-efficacy had a significant negative effect on cognitive load, which in turn positively influenced learning motivation.

**Mediation Analysis:**

Indirect Effect: The relationship between self-efficacy and learning motivation is partially mediated by cognitive load. High self-efficacy reduces cognitive load, which then enhances learning motivation by freeing up cognitive resources for task engagement.

Direct Effect: While self-efficacy directly influences learning motivation, the indirect effect through cognitive load provides a more comprehensive understanding of the mechanisms at play.

10. The literature review needs an in-depth analysis of studies associated with the current research scope.

**Answer:**

Thank you for highlighting the need for a more in-depth literature review. I have taken your feedback seriously and have made several revisions to ensure that the literature review is more comprehensive and aligned with the current research scope. Here are the key changes:

**Expanded Literature Search:**

I have conducted a more extensive search using databases such as Web of Science, Scopus, and Google Scholar to identify relevant studies. This has allowed me to include a broader range of high-quality references.

I have also included recent studies (published within the last 5 years) to ensure that the review is up-to-date and reflects the latest developments in the field.

**Thematic Analysis:**

I have organized the literature review into thematic sections to provide a structured and coherent analysis. The themes include:

**Self-Efficacy and Learning Motivation:**

I have discussed the theoretical foundations of self-efficacy (Bandura, 1997) and its role in predicting learning motivation (Chen et al., 2020).

I have included studies that support the positive relationship between self-efficacy and motivation (e.g., Pajares & Schunk, 2001; Usher & Pajares, 2008).

**Self-Efficacy and Cognitive Load:**

I have explored the relationship between self-efficacy and cognitive load, drawing from cognitive load theory (Sweller, 1988).

I have included studies that show how self-efficacy can reduce cognitive load (Hwang et al., 2021; Wang et al., 2022).

**Cognitive Load and Learning Motivation:**

I have discussed how high cognitive load can negatively impact learning motivation (Sweller, 1988; Ryan & Deci, 2000).

I have included studies that support the mediating role of cognitive load in the relationship between self-efficacy and learning motivation (Zimmerman, 2000; Hwang et al., 2021).

**Critical Evaluation:**

I have critically evaluated the strengths and limitations of the studies included in the review. This includes discussing methodological approaches, sample sizes, and the generalizability of the findings.

I have also highlighted areas of consensus and disagreement in the literature, providing a balanced view of the current state of research.

11. The methodology must be delineated and clarified with more detail and descriptions.

**Answer:**

Thank you for your valuable feedback. I have expanded the methodology section to provide more detailed and clear descriptions.

12. The proposed title is not in concert with the research as the synergy of self-efficacy, motivation, and cognitive load are investigated rather than the absolute mediating effect of cognitive load on the dependent and independent variables.

**Answer:**

The new title is:" Synergy of Self-Efficacy, Cognitive Load, and English Learning Motivation among Vocational College Students"

13. Theoretical implications: how are your research findings and claims in concert with Shao and Kang’s “parent-child relationship” concerns?

**Answer:**

Thank you for raising this important point. My research findings and claims are indeed in concert with Shao and Kang's (2022) concerns regarding the parent-child relationship and its impact on learning engagement. Here’s how:

**Role of Self-Efficacy:**

My Study: I found that self-efficacy plays a crucial role in managing cognitive load and enhancing learning motivation among vocational college students. High self-efficacy reduces cognitive load, which in turn increases learning motivation.

Shao and Kang (2022): They highlight the pivotal role of self-efficacy in the impact of parent-child relationships on learning engagement. Their findings suggest that children with higher self-efficacy are more likely to engage in learning activities, even when faced with challenges.

**Mediating Role of Cognitive Load:**

My Study: Cognitive load is identified as a mediator in the relationship between self-efficacy and learning motivation. Reducing cognitive load through high self-efficacy enhances learning motivation.

Shao and Kang (2022): While they do not explicitly discuss cognitive load, their findings imply that a supportive parent-child relationship can enhance self-efficacy, which in turn can reduce the perceived difficulty of learning tasks, similar to the concept of cognitive load.

**Theoretical Framework:**

My Study: I integrate Social Cognitive Theory (SCT) and Cognitive Load Theory (CLT) to explore the mediating role of cognitive load.

Shao and Kang (2022): They use a self-determination theory perspective to explore the chain mediating roles of learning motivation and academic self-efficacy. Both studies emphasize the importance of self-efficacy in educational outcomes.

By aligning these findings, it becomes evident that self-efficacy is a central construct in both studies, influencing learning outcomes through various mechanisms, including cognitive load and learning motivation.

14. Findings need to be further discussed and compared with similar research findings.

**Answer:**

I have expanded the discussion section to further compare my findings with similar research findings. Here are the key points:

**Comparison with In’am and Sutrisno (2021):**

My Study: I found that self-efficacy positively impacts deep and achievement motivation and negatively influences cognitive load.

In’am and Sutrisno (2021): They highlighted the role of self-efficacy in stimulating learning motivation through cooperative learning models. Both studies support the idea that self-efficacy is a strong predictor of learning motivation.

**Comparison with Evans et al. (2024):**

My Study: Cognitive load is identified as a mediator in the relationship between self-efficacy and learning motivation.

Evans et al. (2024): They explored the relationships between cognitive load and motivation from a self-determination theory perspective. Their findings suggest that cognitive load can significantly impact motivation, aligning with my study’s results.

**Comparison with Wang and Xing (2019):**

My Study: The use of digital textbooks and online platforms can influence cognitive load and learning motivation.

Wang and Xing (2019): They found that the use of digital textbooks on mobile devices can enhance learning outcomes, but the effectiveness depends on how well the cognitive load is managed. This supports the importance of managing cognitive load in digital learning environments.

**Comparison with Dweck (2017):**

My Study: Self-efficacy and cognitive load management are crucial for learning motivation.

Dweck (2017): She emphasizes the role of growth mindset in learning, suggesting that students who believe they can improve through effort are more motivated. This aligns with the idea that high self-efficacy can enhance motivation by reducing cognitive load.

15. Practical implications: AI’s incorporation is not well-chained to the findings and implications of the current study.

**Answer:**

I have revised the practical implications section to better incorporate the findings and implications of the current study, particularly in relation to the use of AI. High self-efficacy reduces cognitive load, enhancing learning motivation. AI tools can help manage cognitive load by adjusting task difficulty based on student performance, reducing cognitive overload and boosting motivation. Additionally, self-efficacy is crucial for enhancing learning motivation, and AI-powered educational platforms can provide personalized feedback and support to build students' self-efficacy. For example, intelligent tutoring systems can offer immediate feedback and encouragement, helping students gain confidence. AI can also promote deep and achievement motivation by creating engaging and interactive learning experiences. Technologies like virtual reality (VR) and augmented reality (AR) can simulate real-world scenarios, making learning more immersive and meaningful. Finally, social self-efficacy improves learning motivation through collaboration, and AI can facilitate this by matching students with peers who have complementary skills and interests. Online platforms can use AI algorithms to form effective study groups and provide tools for collaborative projects.
